# A Rare Case of Rhabdomyolysis

**DOI:** 10.7759/cureus.31519

**Published:** 2022-11-15

**Authors:** Francisco Pombo, Ana Raposo, Marta Dalila Martins, Inês Ferreira, Lindora Pires

**Affiliations:** 1 Internal Medicine, Centro Hospitalar do Tâmega e Sousa, Penafiel, PRT; 2 Rheumatology, Centro Hospitalar do Tâmega e Sousa, Penafiel, PRT

**Keywords:** oral methotrexate, corticosteroids, paresis, necrotizing myositis, myalgia, rhabdomyolysis

## Abstract

Rhabdomyolysis is a pathological condition presenting with symptoms of localized or generalized myalgia and weakness, associated with an increase in serum creatine kinase level and, often leading to myoglobinuria and acute kidney injury. It has a wide range of etiologies. Immune-mediated necrotizing myopathy (IMNM) is a rare type of inflammatory myopathy, that leads to rhabdomyolysis, and it is divided into three different subtypes: anti-3-hydroxy-3-methylglutaryl-coA reductase (anti-HMGCR, anti-signal recognition particle (anti-SRP), and seronegative. There are slight differences in incidence, age of onset, clinical course, and prognosis between these subtypes.

We describe the case of a 67-year-old female with myalgias and weakness of the thighs for six weeks. Laboratory findings showed marked rhabdomyolysis and severe acute kidney injury. The workup led to the diagnosis of seronegative immune-mediated necrotizing myopathy (IMNM) and treatment with corticosteroid and methotrexate was introduced, which led to marked clinical improvement.

## Introduction

Rhabdomyolysis is a pathological condition due to injury of skeletal muscle. It ranges from an asymptomatic illness to a condition presenting with localized or generalized myalgia and weakness, associated with an increase in the serum of muscle enzymes, leading to myoglobinuria and acute kidney injury. It has a wide range of different causes. The most frequent are traumatic injuries, but there are other causes, such as genetic, infectious, toxic, metabolic, ischemic, and inflammatory, and it is a consequence of the dissolution of skeletal muscle and leakage of muscle cell contents, such as creatine kinase, lactate dehydrogenase, aldolase, alanine and aspartate aminotransferase, and electrolytes [1].

Immune-mediated necrotizing myopathy (IMNM) is a type of inflammatory myopathy of autoimmune origin, belonging to the wider group of idiopathic inflammatory myopathies. It is a relatively rare condition, with a prevalence of 9 to 14 cases per 100.000 people [2,3]. Generally, it can be divided into three types, according to the specific antibody in question: anti-3-hydroxy-3-methylglutaryl-coA reductase (anti-HMGCR), anti-signal recognition particle (anti-SRP), and seronegative [4]. The third type is dependent on a positive muscle biopsy to meet diagnostic criteria. The clinical features are characterized by proximal muscle weakness, myalgias, muscle atrophy, and dysphagia. Extra-muscular manifestations are common, specifically interstitial lung disease and myocarditis [4]. It usually has an acute onset, with a small group of patients experiencing slow progressive evolution [4]. There is a frequent association with malignancy (of no particular type), especially in patients with the seronegative type. It results in myofiber necrosis with minimal inflammatory cell infiltrate on muscle biopsy [5].

We report a case of a 67-year-old female of European origin with arterial hypertension, dyslipidemia, and first-degree atrioventricular block. She was medicated with enalapril, lercanidipine, and simvastatin.

## Case presentation

The patient came into the emergency department with myalgia and weakness of the thighs with progressive worsening over six weeks. There was no history of trauma or infection. She also mentioned dark-colored urine. The blood pressure was 132/78 mmHg, pulse was 96 bpm, and respiratory frequency was 18 cpm, with a normal body temperature (36.5ºC). A full neurological examination showed significant proximal weakness of the lower limbs (Medical Research Council [MRC] scale of 2 on flexion). There were no skin abnormalities present and there was no noticeable decrease in weight or overall body muscle mass. The laboratory results showed a marked elevation of muscle enzymes (creatine kinase of >40000 U/L, myoglobin of >4104 ng/mL, and aldolase of 307 U/L), the elevation of liver enzymes (AST of 2065 U/L and ALT of 1278u/L), hyperkalemia (6.8 mmol/L), the elevation of lactate dehydrogenase (1804 U/L), and acute kidney injury (BUN of 89.14 mg/dL and creatinine of 4.85 mg/dL). The urine was of dark red coloration and urinalysis revealed the presence of myoglobinuria. The laboratory results are shown in Table 1. As such, a diagnosis of rhabdomyolysis was reached.

**Table 1:** Laboratory results at admission in the Emergency Department.

	At admission	Reference Range
Creatine kinase	> 40000 U/L	< 149 U/L
Myoglobin	> 4104 ng/mL	14.3 – 65.8 ng/mL
Aldolase	307 U/L	1.2 – 7.6 U/L
Aspartate transaminase	2065 U/L	10 – 31 U/L
Alanine transaminase	1278 U/L	10 – 31 U/L
Alkanine phosphatase	81 U/L	30 – 120 U/L
γ-glutamyltransferase	12 U/L	7 – 32 U/L
Lactate dehydrogenase	2524 U/L	135 – 225 U/L
Blood urea nitrogen	89.14 mg/dL	6 – 24 mg/dL
Creatinine	4.85 mg/dL	0.66 – 1.09 mg/dL
Sodium	137 mmol/L	135-145 mmol/L
Potassium	6.8 mmol/L	3.5 – 5.0 mmol/L
Calcium	4.4 mEq/L	4.4 – 5.3 mEq/L

**Figure 1:**
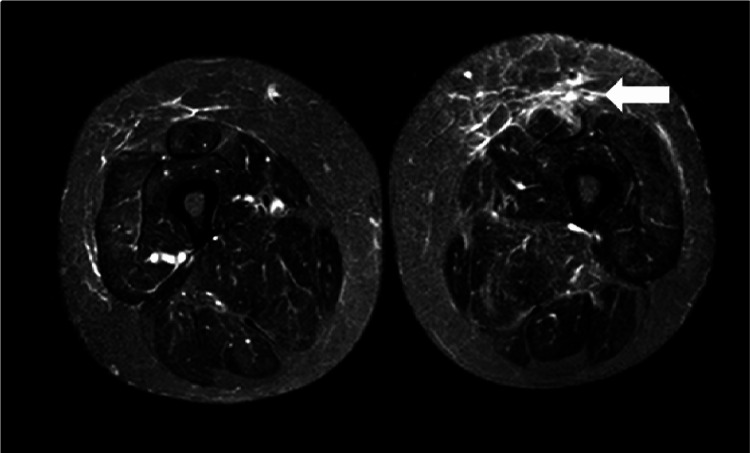
Short TI inversion recovery (STIR) sequence of bilateral thigh magnetic resonance showing generalized muscle edema, muscle atrophy, and fatty replacement.

The patient was admitted to the Internal Medicine department, where vigorous fluid therapy was initiated with a slow recovery of kidney function. The patient had an extensive diagnostic workup. First, infectious causes typically associated with rhabdomyolysis were excluded: HIV, hepatitis B and C, Leptospira, Treponema pallidum, Coxiella burnetti, Mycoplasma pneumoniae, Cytomegalovirus, Epstein-Barr virus, and Coxsackie virus. Secondly, an autoimmunity panel with complement proteins (C3 and C4), immunoglobins (IgA, IgM, and IgG), anti-nuclear and anti-neutrophil cytoplasm antibodies, and a myositis panel (including anti-HMGCR, SRP, cN-1A, Ro52, OJ, EJ, PL12, PL7, Jo1, PM/Scl 75, OM/Scl 100, Ku, SAE1, NXP2, MDA5, TIF1gama, Mi-2beta, and Mi-2alfa) were normal. Electromyography showed complex repetitive discharges, fibrillation potentials, and positive sharp waves in bilateral iliopsoas muscles on spontaneous activity and amplitude and polyphasic potentials with early recruitment in bilateral iliopsoas muscles on voluntary activity, evidence of a myopathic process with muscle necrosis proximally in the lower limbs. A lower limb MRI revealed generalized muscle edema, muscle atrophy, and fatty replacement, especially in the biceps femoris, semimembranosus, and rectus femoris muscles (Figure 1). A muscle biopsy was performed and revealed an inflammatory myopathy with extensive necrosis of muscle fibers and mononuclear infiltrate in the perimysium and endomysium. These findings are indicative of the presence of IMNM. As such, a final diagnosis of seronegative IMNM was reached. Because of the frequent association between IMNM and malignancy, an extensive search of occult malignancy was performed with no significant findings (normal full body CT-scan, skin examination, breast, thyroid and pelvic ultrasounds, esophagogastroduodenoscopy, colonoscopy, and cervical cytology). Also, a high-resolution CT of the chest showed no evidence of interstitial lung disease, a common extra-muscular manifestation associated with seronegative IMNM.

The muscle enzymes decreased considerably during the hospital stay, with the only therapy being fluids and statin eviction. No specific treatment was introduced at this stage. Still, and after discharge, the patient remained symptomatic, with myalgias and muscle weakness. As such, treatment with corticosteroids was introduced (prednisolone 60mg daily for one week followed by slow tapering), with moderate pain and complete weakness recovery (MRC scale of 5). After one month, oral methotrexate was introduced. The patient was also referred to a physical rehabilitation program. It is important to note that after the normalization of serum levels of muscle enzymes, there were no more incidences of increasing trends. We believe this correlates with disease activity improvement rather than widespread necrosis of the muscle since the levels of creatinine and aldolase (1.9 U/L) remained within normal range, indicating normal muscle mass. An MRI will be performed at 3-6 months to monitor response to treatment.

## Discussion

IMNM is a rare condition belonging to the wider group of idiopathic inflammatory myopathies. It has been associated with viral infections, connective tissue diseases, malignancy, and certain toxins [6]. It can be divided into three types: anti-HMGCR, anti-SRP, and seronegative. In this case, its subtype was considered seronegative since both anti-HMGCR, and anti-SRP were negative. There are slight differences in incidence, age of onset, clinical course, and prognosis between the subtypes. In addition, it lacks dermatological findings, typical of dermatomyositis, a common differential diagnosis. Anti-SRP has a slightly higher incidence compared to the other two types. Seronegative IMNM is rare, representing 10-12% of cases of IMNM and, as in the other subtypes, it is more prevalent in females aged 40 to 50 [6]. Onset in the young is associated with a worse prognosis. The 2017 European Neuromuscular Centre criteria divide the diagnosis criteria into three categories: clinical, serological, and muscle biopsy features. The clinical criteria are high creatine kinase and proximal weakness; serological criteria include the presence of anti-HMGCR or anti-SRP antibodies; in the absence of antibodies, muscle biopsy features (necrotic fibers, myophagocytosis, and paucilymphocytic infiltrate) are required. Our patient met both clinical and muscle biopsy criteria, considering it is a seronegative case.

In seropositive IMNM, the antibody titer correlates with disease activity. They target SRP and HMGCR, ubiquitous proteins present in the cell surface. In vitro, these antibodies were associated with high levels of inflammatory cytokines (such as TNF and IL-6) and reactive oxygen species [5]. Anti-SRP autoantibodies are screened by enzyme-linked immunosorbent assay (ELISA) or line blot assay, and anti-HMGCR antibodies are usually screened by ELISA [2].

A muscle MRI is not diagnostic but can help to determine the extension and distribution of the disease and to monitor response to treatment. Usual findings are muscle atrophy, edema, and fatty replacement [2].

Statin use is associated with the anti-HMGCR subtype, distinctly different from statin-induced myalgias and other inflammatory myopathies. In this subtype, the immune system becomes sensitized to increased levels of HMG-CoA reductase that is upregulated by statins, resulting in a necrotizing immune-mediated injury to cells that express this enzyme [7]. In this case, even though the patient was medicated with simvastatin, her specific subtype doesn’t allow for the correlation with statin use.

IMNM is a chronic disease with a notably increased mortality risk that is attributed to malignancy and more commonly myocarditis. Interstitial lung disease can also increase its morbidity. Even though seropositive disease is associated with a more severe course with muscle atrophy and consequent weakness, classically seronegative patients are thought to have a lower survival rate due to its association with malignancy. However, Shelly et al. recently found no evidence of increased malignancy [8]. In our patient, cancer screening showed no evidence of cancer.

There are no randomized controlled trials, published to our knowledge, for the treatment of IMNM. Nevertheless, the therapeutic approach begins with corticosteroids. However, corticosteroid monotherapy is insufficient for disease control in most patients, and as such, most require second-line agents, such as methotrexate, azathioprine, and rituximab. Intravenous immunoglobulins are recommended for patients without adequate control after six months of treatment [4].

We consider this case to be different from other similar cases published of seronegative IMNM, due to the severity of clinical presentation. Our patient presented to the emergency department with severe motor impairment, a very marked elevation of serum levels of muscle enzymes, and severe acute kidney injury, in a subacute evolution (six weeks versus four to five months as in other case reports) [9-12]. The serum levels of muscle enzymes are significant (268 times the normal range for creatine kinase, 63 times for myoglobin, and 40 times for aldolase). This is in contrast with the seropositive subtypes (usually around 30-fold range) [4] and other case reports of the seronegative subtype (40 to 60-fold range for creatine kinase) [10-12]. Also, in this case, myalgia and rhabdomyolysis recovered significantly before any specific treatment began, even though motor recovery only started after corticosteroids and methotrexate. These slight differences on clinical presentation are probably because seronegative INMN is a default denomination, encompassing other yet-to-be-identified subtypes. Finally, is important to note that the association of rhabdomyolysis and statin use in similar cases may be tempting. This case, as others relating to seronegative IMNM, shows that even though the patient was on statins (a very common medication in the general population), the association cannot be made in every case, as proven by the diagnosis of seronegative IMNM.

## Conclusions

This case demonstrates a female patient presenting with severe rhabdomyolysis with proximal muscle weakness and a diagnosis of seronegative IMNM, with no history or evidence of current cancer diagnosis (typically associated with seronegative type) and, at the same type, with a prolonged history of statin use (which is usually associated with anti-HMGCR type and not the seronegative type). This and the fact that it is a rare disease with a considerable list of differential diagnoses illustrates the need for rigorous diagnostic workup for disease and subtype identification, due to its different clinical courses, treatments, and complications.
